# Effect of Polyunsaturated Fatty Acids on Homocysteine Metabolism through Regulating the Gene Expressions Involved in Methionine Metabolism

**DOI:** 10.1155/2013/931626

**Published:** 2013-05-23

**Authors:** Tao Huang, Xiaojie Hu, Nicholas Khan, Jing Yang, Duo Li

**Affiliations:** ^1^Department of Food Science and Nutrition, Zhejiang University, 866 Yu-Hang-Tang Road, Hangzhou 310059, China; ^2^APCNS Centre of Nutrition and Food Safety, Hangzhou, China; ^3^Department of International Health, Johns Hopkins Bloomberg School of Public Health, Baltimore, MD 21205, USA

## Abstract

The objective was to investigate the regulatory effect of polyunsaturated fatty acids (PUFAs) on mRNA expression of key genes involved in homocysteine (Hcy) metabolism. Eighty male Sprague Dawley rats were randomly divided into eight groups. The oils were orally administered daily for 8 weeks. Plasma Hcy, phospholipids fatty acids, and mRNA expression were determined. Compared with the control group, plasma Hcy was significantly decreased in the 22:6*n*-3 and conjugated linoleic acid (CLA) groups; mRNA expression of *Mthfr* was significantly upregulated in the 22:6*n*-3, 20:5*n*-3, and 18:3*n*-3 groups and downregulated in the 18:2*n*-6 and stearolic acid (SO) groups. *Mat1a* was upregulated in the 22:6*n*-3, 20:5*n*-3, 18:3*n*-3, and CLA groups. In addition, *Cbs* was upregulated in the 22:6*n*-3, 20:5*n*-3, 18:3*n*-3 and CLA groups while downregulated in 18:2*n*-6 and SO groups. Dietary 22:6*n*-3 and CLA decrease the plasma concentration of Hcy. mRNA expression of *Mthfr, Mat1a, Cbs* and *Pemt, Gnmt, Mtrr*, and *Bad* is upregulated by *n*-3 PUFA and downregulated by *n*-6 PUFA. CLA upregulates mRNA expression of *Mat1a* and *Cbs*.

## 1. Introduction

Homocysteine (Hcy) is a sulfur-containing amino acid and a by-product of methionine metabolism [1]. Elevated plasma Hcy has been implicated as an independent risk factor for cardiovascular disease (CVD) [2].

Intracellular Hcy can be irreversibly degraded to cysteine through the transsulfuration pathway, a process primarily found in cells of the liver and kidneys. Two enzymes in this pathway, cystathionine b-synthase (CBS) and cystathionine gamma-lyase (CSE), are dependent on pyridoxal-5-phosphate, a biologically active form of vitamin B_6_, as a cofactor. Hcy can also be remethylated to methionine by the enzyme methionine synthase (MS). This enzyme uses methylcobalamin, a biologically active form of vitamin B_12_, as a cofactor. The methyl group for the latter reaction is donated by 5-methyl-tetrahydrofolate (5-methyl-THF). This form of folate is produced by the enzyme 5,10-methylenetetrahydrofolate reductase (MTHFR), which in turn uses flavin adenine dinucleotide, a biologically active form of vitamin B_2_, as a cofactor. In most cases, disturbances in intracellular Hcy metabolism leads to elevated Hcy concentrations. Genetically determined functional deficiencies in enzymes integral to Hcy metabolism, like CBS, have a significant impact on Hcy concentration. 

Numerous studies have investigated the effects of environmental factors on plasma Hcy metabolism since McCully hypothesized that elevated Hcy in blood was a risk factor for atherosclerosis [3]; some recent systematic reviews have described the impact of lifestyle and dietary factors on levels of plasma Hcy [1, 4]. Dietary intake of fish oil rich in *n*-3 polyunsaturated fatty acid (PUFA) leads to increased levels of *n*-3 PUFA in tissues [5, 6], which is associated with a protective effect on the cardiovascular system. We have previously demonstrated that increased total *n*-3 PUFA and *n*-3/*n*-6 PUFA in platelet phospholipids (PL) is associated with decreased plasma Hcy [7, 8]. One study demonstrated that *n*-3 PUFA supplementation decreases plasma Hcy in diabetic dyslipidemia treated with a statin-fibrate combination [9]. Further, plasma Hcy concentration is significantly decreased in acute myocardial infarction patients after one year of *n*-3 PUFA treatment [10]. Based on the previous inconsistent results from the intervention studies, our meta-analysis demonstrated that high consumption of *n*-3 PUFA decreases plasma Hcy concentration [11].

Our animal study suggested that 22:6*n*-3 decreases plasma Hcy concentration through regulating critical gene expression and enzyme activity [12]. Our population studies found that dietary fatty acids interact with methylenetetrahydrofolate reductase (*MTHFR*) and methionine adenosyltransferase I, alpha (*MAT1A*) genetic variants in determining plasma Hcy concentration [13, 14]. Recently an intervention study also demonstrated that consumption of *n*-3 PUFA supplements (3 g/day) for 2 months decreases the levels of Hcy in those with type 2 diabetes mellitus [15].

However, the mechanism by which *n*-3 PUFA and plasma Hcy levels are associated is not yet fully understood. Therefore, to explore the potential mechanisms by which PUFA might regulate Hcy metabolism, we conducted an animal study to investigate the regulatory effects of different PUFA-rich oils on the mRNA expression of key genes involved in Hcy metabolism. 

## 2. Materials and Methods

### 2.1. Animal Study Design

All procedures were approved by the Ethics Committee of the College of Biosystems Engineering & Food Science at Zhejiang University. Eighty male Sprague Dawley (SD) rats aged 3 weeks, weighing 120 ± 10 g, were purchased from the Zhejiang Laboratory Animal Center (Hangzhou, China). The rats were housed in a room under a 12/12 h light/dark cycle at 22°C. Two weeks later, the rats were randomly divided into eight groups of 10 rats (*n*-10 each group) and were fed a regular diet supplemented with different oils: a control group with no oil supplements (CC); an olive oil group (18:1) fed olive oil (0.5 mL); a tuna oil group (22:6*n*-3) fed a mix of oils (tuna oil 100 mg/kg BW + olive oil = 0.5 mL); a salmon oil group (20:5*n*-3) fed a mix of oils (salmon oil 100 mg/kg BW + olive oil = 0.5 mL); a group fed linseed oil (18:3*n*-3) (linseed oil 100 mg/kg BW + olive oil = 0.5 mL); a group fed corn oil (18:2*n*-6) (corn oil 100 mg/kg BW + olive oil = 0.5 mL); a group fed conjugated linoleic acid (CLA) oil (conjugated linoleic acid oil 100 mg/kg BW + olive oil = 0.5 mL); a group fed stearolic acid (SO) (stearolic acid oil 100 mg/kg BW + olive oil = 0.5 mL). 

All rats were allowed free access to water and regular diet. The oil was orally administered using a stomach tube every day. The rats were killed at eight weeks, and blood (5 mL) was drawn from the abdominal vein. The liver and lungs were rapidly removed, weighed, frozen in liquid nitrogen, and stored at −70°C. 

### 2.2. Plasma Measurements

The blood was centrifuged at 2000 rpm for 15 minutes, and the plasma was collected. Total plasma Hcy was determined by polarized fluorescence immunoassay in an AXSYM system [16]. Plasma folate and vitamin B_12_ were measured using immulite chemiluminescent kits according to the manufacturer's instructions (Diagnostic Products Corporation/Siemens, Los Angeles, CA).

### 2.3. Assay of Phospholipids *n*-3 PUFA in Tissues

Total lipid content of plasma was extracted with solvents, the PL fraction was separated by thin lay chromatography (TLC), and the fatty acid methyl esters were prepared and separated by gas-liquid chromatography as previously described [17].

### 2.4. Assay of mRNA Expression of Critical Genes Involved in Methionine Metabolism

#### 2.4.1. Quantitative Real Time PCR Measurements

Total RNA from the livers was extracted using the Trizol reagent (Shingene, Shanghai, China). The first strand cDNA was synthesized using cDNA synthesis kit (Shinegene, Shanghai, China), the Real Time PCR were conducted on iCycler PCR using the HotStart DNA Master SYBR Green I kit (Takara, Dalian, China) [18]. Primers used are listed in Table 1. All PCR tests were carried out in duplicate with a final volume of 20 *μ*L containing cDNA. Thermal cycling conditions were set as follows: an initial DNA denaturation step at 95°C for 5 seconds, followed by 40 cycles of denaturation at 95°C for 5 seconds, primer annealing at optimal temperature for 20 s, extension at 72°C for 30 s, and an additional incubation step at 80–85°C for 30 s to measure SYBR Green I fluorescence. Finally, melt curve analysis was performed by slowly cooling the PCR from 95 to 60°C (0.5°C per cycle) with simultaneous measurement of the SYBR Green I signal intensity. Gene expression was quantified using the comparative C(t) method [18]. 

### 2.5. Statistical Analysis

The data analyses were performed using an SPSS version 12 (SPSS Inc., Chicago, IL, USA) software program. All data are expressed as mean ± SD. Statistical analysis was performed using one-way ANOVA; differences between treatments were considered to be statistically significant at *P* < 0.05. 

## 3. Results

### 3.1. The Effects of Oils on Plasma Phospholipids Fatty Acids Composition in Rats

After 8 weeks of treatment, plasma PL 22:6*n*-3, 20:5*n*-3, CLA, 18:2*n*-6, and 18:3*n*-3 were significantly increased in groups supplemented with 22:6*n*-3, 20:5*n*-3, CLA, 18:2*n*-6, and 18:3*n*-3 oils, respectively (Table 2).

### 3.2. The Effects of Oils on Plasma Biochemical Measurements in Rats

Plasma glucose and uric acid were significantly decreased, while total protein was significantly increased in the 22:6*n*-3 group. Plasma low-density lipoprotein (LDL), total cholesterol (TC), glucose, and aspartate aminotransferase (AST) were significantly decreased in the 20:5*n*-3 group. Plasma HDL was significantly increased, while plasma glucose was decreased in the 18:3*n*-3 group (Table 3). 

### 3.3. The Effects of Oils on Plasma Hcy and Vitamin B_12_ and Folate in Rats

**Figure 1:**
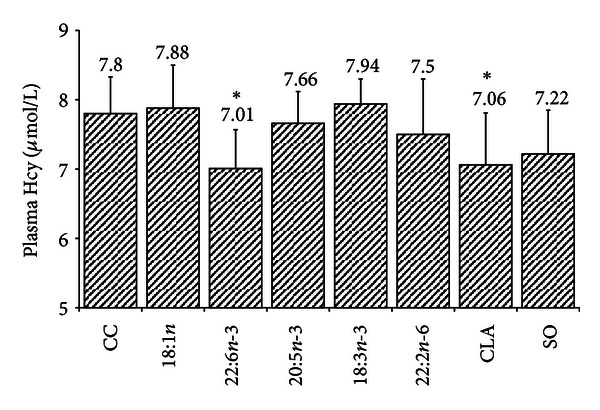
The effect of fatty acids on plasma Hcy concentration. *n*-10, data was expressed as mean ± SD. *indicate significant difference when compared with 18 : 1 group. CC: control group; SO: stearolic acid.

The oils differential effects on plasma Hcy concentration in rats. Plasma Hcy concentration was significantly decreased in the 22:6*n*-3 and CLA groups, while plasma Hcy concentration was not significantly affected in other groups (Figure 1). In addition, plasma vitamin B_12_ and folate concentration were not significantly affected in treatment groups (Table 2).

### 3.4. Effects of Oil on the Expression of Critical Genes Involved in Hepatic Hcy Metabolism

mRNA expression of *Mthfr* was significantly upregulated in the 22:6*n*-3, 20:5*n*-3, and 18:3*n*-3 groups and down-regulated in the 18:2*n*-6 and SO groups.* Bhmt* was significantly upregulated in the 20:5*n*-3, 18:3*n*-3, and 18:2*n*-6 groups. *Mat1a *was significantly upregulated in the 22:6*n*-3, 20:5*n*-3, 18:3*n*-3, and CLA groups. In addition, *Cbs *was significantly upregulated in the 22:6*n*-3, 20:5*n*-3, 18:3*n*-3, and CLA groups, while down-regulated in the 18:2*n*-6 and SO groups. *Pemt* was significantly upregulated in the 20:5*n*-3 and 18:3*n*-3 groups, and downregulated in the SO group. However, the different oil supplements did not significantly affect the mRNA expression of *Sahh*. Other critical gene expression (*Bhmt, Cse, Chka, Tyms, Chdh, Gnmt, Mthfs, Mtr, Cept1, Mtrr, Etnk1,* and *Bad*) involved in Hcy and phospholipids metabolism was also regulated by the different oils (Table 4). 

## 4. Discussion

In the present study, we found that oils rich in 22:6*n*-3 and CLA significantly decreased plasma Hcy concentration. These oils have different regulatory effects on the mRNA expression of a critical gene involved in Hcy metabolic pathway.

**Figure 2:**
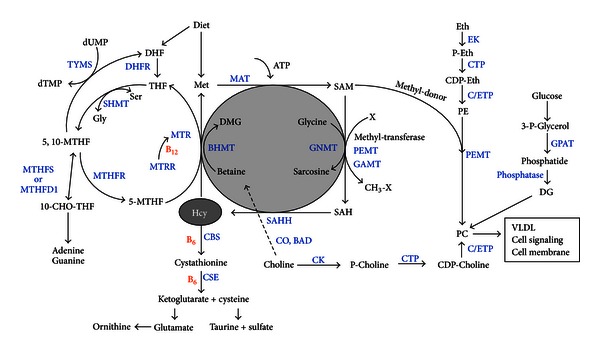
Homocysteine metabolism. *Mthfr*:methylenetetrahydrofolate reductase; *Sahh*:S-adenosylhomocysteine hydrolase; *Bhmt*:betaine-homocysteine methyltransferase; *Cbs*: cystathionine beta synthase; *Mat1a*: methionine adenosyltransferase I, alpha;* Pemt*: phosphatidylethanolamine N-methyltransferase; *Cse*: cystathionine gamma-lyase;* Chka*: choline kinase alpha; *Tyms*: thymidylate synthetase; *Chdh*: choline dehydrogenase; *Gnmt*: glycine N-methyltransferase; *Mthfs*: 5,10-methenyltetrahydrofolate synthetase; *Mtr*: 5-methyltetrahydrofolate-homocysteine methyltransferase; *Cept1,* choline/ethanolamine phosphotransferase 1; *Mtrr*: 5-methyltetrahydrofolate-homocysteine methyltransferase reductase; *Bad*: betaine aldehyde dehydrogenase; *Etnk1*: ethanolamine kinase 1.

*n*-3 PUFAs are essential for normal growth and development and for the prevention and treatment of coronary heart disease [19, 20], hypertension [21], and type 2 diabetes mellitus [22, 23]. Previous studies suggested that *n*-3 PUFAs play an important role in Hcy metabolism [13, 14]. The possible mechanism by which *n*-3 PUFA regulate Hcy metabolism was also investigated [12, 24]; Piolot reported the apparent interaction of *n*-3 PUFA and NO on Hcy metabolism in healthy people [24]. They suggested a probable mechanism by which *n*-3 PUFA supplementation can reduce the production of Hcy. The reduced Hcy concentrations observed in their study are attributed to possible oxidative stress induction by *n*-3 PUFA and stimulation of the oxidative catabolism of Hcy [25]. Investigators have previously reported increased susceptibility to oxidative stress due to *n*-3 PUFA supplementation [26]. Our animal and population studies have also investigated the potential mechanisms [12–14] (Figure 2). In our animal study, we found that plasma Hcy was significantly decreased by tuna oil rich in 22:6*n*-3. MAT activity was significantly increased, and MAT mRNA expression was significantly upregulated by 22:6*n*-3; CSE mRNA expression was significantly upregulated by 22:6*n*-3. We have suggested that 22:6*n*-3 decreases the concentration of Hcy despite increasing MAT activity and upregulation of MAT mRNA expression through compensatory CSE mRNA expression, both of which are involved in Hcy metabolism [12]. 

However, the determination of Hcy concentration is multifactorial shaped by gene-environment interactions. Thus, genetic variants involved in the Hcy metabolic pathway may modify the effect of dietary fatty acids on plasma Hcy in humans. Our population studies found an interaction between dietary fatty acids and genetic variants *MTHFR* and *MAT1A* in determining plasma Hcy concentration [13, 14]. Two functional *MTHFR* variants, 1298A > C and 677C > T, which were not in linkage disequilibrium, were significantly associated with hypertension. Importantly, the variants exhibited significant interactions with intakes of total and *n*-6 PUFA and a dietary *n*-3 : *n*-6 PUFA ratio in determining plasma Hcy concentration. In addition, participants with combined genotypes of both SNP (677 TT with 1298 AC or CC) who consumed high levels of *n*-3 PUFA (>0.66% energy) had lower plasma Hcy compared with those who had the same genotype and consumed low levels of *n*-3 PUFA (</=0.66% energy). It was thus suggested that dietary PUFA intake modulates the effect of *MTHFR *variants on plasma Hcy [14]. Moreover, genetic variant *MAT1A* 3U1510 displayed a significant interaction with dietary *n*-3 : *n*-6 PUFA ratio in determining plasma Hcy. 3U1510G homozygotes had significantly lower plasma Hcy concentration than major allele homozygotes and heterozygotes (AA + AG) when the *n*-3 : *n*-6 ratio was >0.09. Two other *MAT1A* variants (d18777 and i15752) also showed significant interactions with different constituents of dietary fat in influencing Hcy concentration. Further, haplotypes consisting of three variants displayed a strong interaction with one's *n*-3 : *n*-6 ratio in influencing Hcy concentrations [13].

Taken together, the studies by Noga et al. [27] and Jacobs et al. [28] provide strong evidence that phosphatidylethanolamine N-methyltransferase (PEMT) plays a significant role in the regulation of plasma Hcy concentrations. Noga et al. utilized PEMT-/- knockout mice and McArdle RH-7777 cells transfected with PEMT to study the contribution of PEMT to Hcy levels. Plasma Hcy concentrations in the Pemt-/- knockout mice were 50% of those in the wild-type mice. Furthermore, when McArdle RH-7777 cells with negligible PEMT activity were transfected to stably express PEMT, there was more than a 2-fold increase in Hcy secretion compared with cells that were transfected with the vector alone. These studies illustrate the potential for PEMT to regulate plasma Hcy or to be a major determinant of Hcy concentration [29]. The PEMT pathway primarily contains PUFA, such as 20:4*n*-6 and 22:6*n*-3 [30], but PEMT also has an important role in the distribution of fatty acids to tissues [30]. An animal study showed that 22:6*n*-3 in plasma phosphatidylcholine may be a potential marker for in vivo PEMT activity in humans [30]. We try to examine the effect of fatty acids on gene expression of *Pemt* in the present study, but did not observe a significant regulatory effect of 22:6*n*-3 on *Pemt *expression, though 20:5*n*-3 and 18:3*n*-3 significantly upregulated the expression of *Pemt*. A recent paper by Ratnam et al. presented convincing evidence that betaine-homocysteine S- methyltransferase (BHMT) plays an essential role in lowering plasma Hcy levels in a diabetic state [31]. Jacobs et al. noted that there was increased activity and abundance of BHMT and MAT, leaving the possibility that BHMT would be able to compensate for the increased Hcy production [28]. The present study showed that *Bhmt* was significantly upregulated in the 20:5*n*-3, 18:3*n*-3, and 18:2*n*-6 groups.

However, considering the significance of *n*-3 PUFA in decreasing plasma Hcy levels and protecting from CVD, more cell cultures, animal studies, and population studies should be conducted to investigate the potential mechanism by which *n*-3 PUFA decreases plasma Hcy concentration. In addition, the genetic variation of some critical genes among populations could account for the difference in the results of intervention studies; nutrigenomics may be essential in identifying interactions between the genetic variants and dietary fatty acids in determining plasma Hcy concentration. 

In summary, dietary intake of 22:6*n*-3 and CLA decrease the plasma concentration of Hcy. Different PUFAs have different effects on the mRNA expression of key genes involved in Hcy metabolism; *n*-3 PUFA upregulates expression, while *n*-6 PUFA down-regulates mRNA expression of *Mthfr*, *Mat1a, Cbs, Pemt, Gnmt, Mtrr,* and *Bad*. *n*-3 PUFA and CLA down-regulate mRNA expression of *Cept1, Etnk1*, and CLA upregulates mRNA expression of* Mat1a *and* Cbs.*

## Figures and Tables

**Table 1:** The primers used in real time PCR.

GenBank Acc.	Symbol	Primer	
NM_030850.1	*Bhmt *	F: TGCGCAGTGCGTTTGGTAA	R: AGGGCGTGGCTCATCAAGTAAG
NM_019179.1	*Tyms *	F: AAGAATCATCATGTGTGCCTGGAA	R: TCCTGACCGCTGGTAAAGCTG
NM_017201.1	*Sahh *	F: TGTGGACCCACCCAGACAAATA	R: CAGCTTGGTCAGCTTGACGTTC
NM_017127.1	*Chka *	F: GGACCAGTTCCACATCAGTGTCA	R: GAGGTTCATCACCAACACTGGCTA
NM_017084.1	*Gnmt *	F: GCCAGCGACAAGATGCTGAA	R: GACAGCGTCAAAGCCATCTCC
NM_017074.1	*Cse *	F: CAGTGATGTTGTCATGGGCTTAGTG	R: CATCCGGATCTGCAGTGTCTTC
NM_013003.1	*Pemt *	F: AGGAGTCCAGAGTGACCACATTTC	R: CACGTAGACGAGTGCCACCA
NM_012860.2	*Mat1a *	F: CCGAGCGAGAGCTACTAGAGGTTG	R: CCGAAATGACCATAGCATGCAG
NM_012522.2	*Cbs *	F: TGCATTATCGTGATGCCTGAGAA	R: GGGAATCGAATCTGGCGTTG
NM_001107894.1	*Etnk1 *	F: TGAGTTTATCCAGGGTGAAGCTTTG	R: TCCAGCCATTGTGTGCATGA
NM_001039003.1	*Mtrr *	F: CAAAGTATGTGCAAGACAACCTCCA	R: TGATTTCTACAAGGGCGTCGTG
NM_001009349.1	*Mthfs *	F: AATCACATGGACATGGTGAGGCTA	R: CAGCCGGTTGCCATCTTTG
NM_001007699.1	*Cept1 *	F: TGCAAGGATACTGGGAATGGCTA	R: AGGCGATGTAAGCCCACAGAG
NM_198731.2	*Chdh *	F: CATCCCTGTGGTGTGCCATC	R: TGTGTGCAAGCATGCTGAATGTA
NM_030864.1	*Mtr *	F: ACTTGCGCAAACTCCGCTATG	R: TGCCAAGGATTCTGTCAACCTG
NM_022698.1	*Bad *	F: GGCAGCCAATAACAGTCATCA	R: GGTACGAACTGTGGCGACTC
XM_342975.4	*Mthfr *	F: AGCTTGAAGCCACCTGGACTGTAT	R: AGACTAGCGTTGCTGGGTTTCAGA
NM_031144.2	*Actb *	F: GGAGATTACTGCCCTGGCTCCTA	R: GACTCATCGTACTCCTGCTTGCTG

*Mthfr*: methylenetetrahydrofolate reductase; *Sahh*: S-adenosylhomocysteine hydrolase; *Bhmt*: betaine-homocysteine methyltransferase; *Cbs*: cystathionine beta synthase; *Mat1a*: methionine adenosyltransferase I, alpha; *Pemt*: phosphatidylethanolamine N-methyltransferase; *Cse*: cystathionine gamma-lyase; *Chka:* choline kinase alpha; *Tyms:* thymidylate synthetase; *Chdh:* choline dehydrogenase; *Gnmt:* glycine N-methyltransferase; *Mthfs:* 5,10-methenyltetrahydrofolate synthetase; *Mtr:* 5-methyltetrahydrofolate-homocysteine methyltransferase; *Cept1:* choline/ethanolamine phosphotransferase 1; *Mtrr:* 5-methyltetrahydrofolate-homocysteine methyltransferase reductase; *Bad:* betaine aldehyde dehydrogenase; *Etnk1:* ethanolamine kinase 1.

**Table 2:** Changes of plasma phospholipids fatty acids in rats after supplementations.

PL fatty acids, %	CC	18:1	22:6*n*-3	20:5*n*-3	18:3*n*-3	18:2*n*-6	CLA	SO
18:1*n*-9	6.62 ± 0.32	10.40 ± 0.34	7.84 ± 0.31	8.30 ± 0.32	7.93 ± 0.36	11.21 ± 0.31	10.50 ± 0.30	9.84 ± 0.31
18:1*n*-7	1.06 ± 0.67	1.12 ± 1.69	0.91 ± 0.66	1.05 ± 0.67	0.77 ± 0.71	0.92 ± 0.22	0.92 ± 0.21	0.91 ± 0.16
18:2*n*-6	27.89 ± 3.87	26.63 ± 3.89	24.22 ± 3.86	27.33 ± 3.87	24.82 ± 3.91	28.29 ± 3.50	32.94 ± 3.49	28.52 ± 2.86
18:3*n*-6	0.82 ± 0.12	1.13 ± 0.16	2.01 ± 0.13	1.52 ± 0.14	0.90 ± 0.23	0.65 ± 0.14	0.48 ± 0.13	0.91 ± 0.13
18:3*n*-3	0.83 ± 0.16	0.96 ± 0.08	0.90 ± 0.05	0.83 ± 0.06	1.23 ± 0.19	0.93 ± 0.07	1.12 ± 0.07	0.90 ± 0.15
20:00	1.12 ± 0.11	1.03 ± 0.16	2.13 ± 0.13	1.78 ± 0.14	0.93 ± 0.23	0.77 ± 0.14	0.15 ± 0.17	1.13 ± 0.13
20:1*n*-9	0.30 ± 0.17	0.28 ± 0.19	0.38 ± 0.16	0.50 ± 0.17	0.25 ± 0.21	0.24 ± 0.17	0.51 ± 0.16	0.31 ± 0.16
20:2*n*-6	0.82 ± 0.61	0.86 ± 0.62	1.78 ± 0.59	0.98 ± 0.6	0.71 ± 0.69	0.55 ± 0.56	0.39 ± 0.55	0.78 ± 0.25
20:3*n*-6	0.30 ± 0.99	0.30 ± 2.01	0.37 ± 1.98	0.30 ± 1.99	0.25 ± 0.08	0.19 ± 1.81	0.35 ± 1.80	0.77 ± 1.91
20:4*n*-6	29.11 ± 3.80	25.43 ± 3.83	22.78 ± 3.81	22.75 ± 3.8	27.08 ± 3.91	23.53 ± 3.45	20.11 ± 3.44	24.78 ± 4.81
20:5*n*-3	0.89 ± 2.87	0.64 ± 2.90	1.20 ± 2.87	1.61 ± 2.87	0.71 ± 2.97	0.29 ± 0.61	0.17 ± 2.60	1.21 ± 0.92
22:1*n*-9	1.13 ± 1.03	0.89 ± 1.06	0.80 ± 1.03	1.98 ± 1.03	0.99 ± 0.13	0.48 ± 0.95	1.51 ± 0.94	0.97 ± 0.13
22:2*n*-6	0.89 ± 0.25	0.64 ± 0.30	1.33 ± 0.27	1.98 ± 0.25	0.99 ± 0.27	0.48 ± 0.27	1.51 ± 0.26	1.13 ± 0.21
22:4*n*-6	0.39 ± 0.52	0.37 ± 0.57	0.52 ± 0.54	0.43 ± 0.53	0.78 ± 0.52	0.18 ± 0.51	0.28 ± 0.50	0.42 ± 0.55
22:5*n*-6	0.42 ± 0.27	0.32 ± 0.31	0.90 ± 0.28	0.91 ± 0.27	0.66 ± 0.21	0.17 ± 0.28	0.14 ± 0.27	0.72 ± 0.27
22:5*n*-3	0.83 ± 0.51	0.71 ± 0.63	0.56 ± 0.62	0.80 ± 0.59	0.71 ± 0.58	0.27 ± 0.57	0.78 ± 0.56	0.52 ± 0.72
22:6*n*-3	3.86 ± 1.42	4.53 ± 1.44	5.87 ± 1.41	5.36 ± 1.4	5.27 ± 1.39	3.47 ± 1.30	4.76 ± 1.29	5.81 ± 2.40
SFA	3.46 ± 3.23	24.29 ± 3.67	24.96 ± 3.64	28.13 ± 3.63	25.07 ± 3.62	26.27 ± 3.30	27.57 ± 3.29	24.67 ± 5.67
MUFA	9.53 ± 5.38	9.53 ± 5.33	13.15 ± 5.33	10.77 ± 5.28	12.42 ± 5.28	10.60 ± 4.80	13.90 ± 4.79	13.25 ± 2.13
PUFA	67.07 ± 4.61	62.52 ± 4.69	62.43 ± 4.66	64.49 ± 4.65	64.13 ± 4.61	59.01 ± 4.22	63.03 ± 4.21	64.41 ± 6.61
*n*-3 PUFA	6.42 ± 3.71	6.85 ± 3.25	8.51 ± 3.22	8.30 ± 3.21	7.93 ± 3.2	4.96 ± 2.93	6.84 ± 2.92	7.51 ± 1.21
*n*-6 PUFA	60.64 ± 3.41	55.68 ± 3.95	53.91 ± 3.92	56.19 ± 3.91	56.19 ± 3.9	54.05 ± 3.56	56.19 ± 3.55	54.91 ± 5.92
*n*-3:*n*-6	0.11 ± 0.09	0.12 ± 0.09	0.16 ± 0.06	0.15 ± 0.05	0.14 ± 0.12	0.09 ± 0.08	0.12 ± 0.07	0.14 ± 0.16

Sample size in each group: *n* = 10, data was expressed as mean ± SD. CC: control group; CLA: conjugated linoleic acid; SO: stearolic acid. SFA: saturated fatty acids, MUFA: monounsaturated fatty acids, PUFA: polyunsaturated fatty acids; PL: phospholipids.

**Table 3:** Changes of biochemical measurements in the rats after the supplementation.

	CC	18:1	22:6*n*-3	20:5*n*-3	18:3*n*-3	18:2*n*-6	CLA	SO
LDL-C, mmol/L	0.22 ± 0.12	0.21 ± 0.11	0.21 ± 0.12	0.18 ± 0.10*	0.20 ± 0.23	0.26 ± 0.11	0.19 ± 0.12	0.28 ± 0.13
TG, mmol/L	0.91 ± 0.76	1.07 ± 0.70	1.07 ± 0.75	1.08 ± 0.74	1.04 ± 0.87	0.73 ± 0.71*	1.06 ± 0.73	0.86 ± 0.84
HDL-C, mmol/L	0.98 ± 0.69	0.98 ± 0.63	1.00 ± 0.68	0.94 ± 0.67	1.11 ± 0.80*	0.98 ± 0.64	0.86 ± 0.67	0.96 ± 0.76
TC, mmol/L	1.82 ± 0.78	1.83 ± 0.72	1.84 ± 0.77	1.72 ± 0.75*	1.94 ± 0.89	1.82 ± 0.73	1.65 ± 0.75	1.80 ± 0.86
Glucose, mmol/L	5.98 ± 2.18	6.01 ± 2.01	5.49 ± 2.16*	4.94 ± 2.14*	4.82 ± 2.27*	5.44 ± 2.03	6.19 ± 2.11	5.15 ± 2.40*
Uric acid, *µ*mol/L	49.02 ± 8.68	44.88 ± 7.99	39.34 ± 8.59*	43.00 ± 8.57	52.12 ± 8.71	48.50 ± 8.08	44.64 ± 8.39	43.92 ± 9.55
ALT,U/L	55.80 ± 9.12	61.20 ± 8.39	56.57 ± 9.03	49.80 ± 9.01	57.60 ± 9.12	58.80 ± 8.49	58.60 ± 8.82	47.40 ± 10.03*
AST, U/L	184.20 ± 26.74	231.40 ± 21.60	168.86 ± 26.48	159.40 ± 26.45*	181.20 ± 26.56	192.40 ± 24.89	190.00 ± 25.86	140.80 ± 29.41*
Creatinine, *µ*mol/L	63.00 ± 10.21	61.78 ± 9.39	63.21 ± 10.11	59.30 ± 10.09*	62.90 ± 10.20	59.78 ± 9.51	57.34 ± 9.87*	56.58 ± 11.23*
BUN, mmol/L	5.95 ± 2.19	6.33 ± 2.01	6.50 ± 2.17	5.81 ± 2.15	5.85 ± 2.26	6.08 ± 2.04	5.83 ± 2.12	5.52 ± 2.41*
Alb, g/L	45.34 ± 9.19	46.12 ± 8.45	45.70 ± 9.10	45.72 ± 9.08	46.22 ± 9.21	45.56 ± 8.56	45.64 ± 8.89	44.60 ± 10.11
Glob, g/L	24.50 ± 7.12	23.20 ± 6.55	27.30 ± 7.05	22.24 ± 7.03*	28.26 ± 7.14	24.00 ± 6.63	23.96 ± 6.89	24.54 ± 7.83
A : G	1.87 ± 0.78	2.00 ± 0.72	1.69 ± 0.77	2.06 ± 0.75	1.67 ± 0.89	1.91 ± 0.73	1.91 ± 0.75	1.84 ± 0.86
TP, g/L	69.84 ± 9.91	69.32 ± 9.12	73.00 ± 9.81*	67.96 ± 9.79	74.48 ± 9.90*	69.56 ± 9.23	69.60 ± 9.58	69.14 ± 10.90
VitaminB_12_, ng/mL	688 ± 78	691 ± 89	690 ± 101	695 ± 70	691 ± 84	690 ± 120	690 ± 97	688 ± 89
Folate, pmol/L	26.8 ± 7.9	26.3 ± 6.1	27.0 ± 5.8	26.9 ± 6.8	27.1 ± 7.3	26.2 ± 5.7	26.0 ± 8.5	26.5 ± 7.1

Sample size in each group: *n* = 10, data was expressed as mean ± SD.

*indicates significant difference when compared with 18:1 group.

CC: control group; CLA: conjugated linoleic acid; SO: stearolic acid; TC: total cholesterol; TG: total triglyceride; HDL-C: high density cholesterol; LDL-C: low density cholesterol; A : G: Alb : Glob; ALT: alanine transaminase; AST: aspartate aminotransferase; TP: total protein; BUN: blood urea nitrogen.

**Table 4:** Gene expression of the critical genes involved in Hcy and phospholipids metabolism pathway.

Gene	CC	18:1	22:6*n*-3	20:5*n*-3	18:3*n*-3	18:2*n*-6	CLA	SO
*Mthfr *	1.0	1.1	**2.4 **	**2.2 **	**2.9 **	**0.3 **	1.1	0.4
*Sahh *	1.0	**0.5 **	0.8	0.9	0.8	0.8	1.3	0.8
*Bhmt *	1.0	0.8	1.1	**2.1 **	**2.7 **	**3.2 **	1.9	0.8
*Mat1a *	1.0	1.1	**2.0 **	**2.0 **	**2.6 **	0.6	**2.3 **	0.7
*Cbs *	1.0	1.6	**2.3 **	**2.6 **	**4.2 **	**0.5 **	**2.2 **	0.5
*Pemt *	1.0	1.5	1.7	**2.7 **	**4.1 **	0.8	1.8	0.4
*Cse *	1.0	0.7	1.5	1.2	0.9	0.6	0.6	0.8
*Chka *	1.0	1.0	1.4	**3.4**	**3.2**	1.2	1.4	**0.2**
*Tyms *	1.0	0.6	1.2	0.6	1.2	**0.2**	0.6	**0.2**
*Chdh *	1.0	0.8	0.6	1.1	1.0	**0.1**	1.1	**0.2**
*Gnmt *	1.0	0.8	1.8	**2.4**	**3.3**	0.6	1.4	**0.3**
*Mthfs *	1.0	0.9	1.4	1.8	**2.9**	1.0	1.6	**0.3**
*Mtr *	1.0	0.9	0.9	**2.6**	**2.1**	0.8	1.0	0.3
*Cept1 *	1.0	1.1	**0.3**	**0.3**	**0.3**	0.6	**0.2**	**0.1**
*Mtrr *	1.0	0.7	0.7	**2.1**	**2.4**	0.7	1.7	0.5
*Etnk1 *	1.0	0.9	**0.3**	**0.4**	0.6	0.7	**0.4**	**0.1**
*Bad *	1.0	0.6	0.7	**2.8**	**3.5**	0.7	1.4	**0.3**
